# Supplementary material for: Sustainable Pest Management Using Novel Nanoemulsions of Honeysuckle and Patchouli Essential Oils against the West Nile Virus Vector, Culex pipiens, under Laboratory and Field Conditions
Source: Plants (Basel). 2023 Oct 25;12(21):3682. doi: 10.3390/plants12213682 (PMC10650709; doi:10.3390/plants12213682)

Table S1. Kruskal–Walli's test for larval mosquito mortality (%) of plant oil groups

Oil groups	Mortality % (mean±SD)					
	0.0	3	6	12	24	48
Low	0±0	25.28±2.79	40.16±3.76	56.64±4.34	70.24±3.58	86.40±3.48
Medium	0±0	34.51±1.08	57.03±2.07	78.29±1.84	88.23±1.15	97.14±1.33
High	0±0	46.40±3.08	72.40±3.37	89.60±2.29	97.60±1.42	100.00±0.00
Chi-square	0.0	21.15	24.15	27.44	29.16	32.15
df	2	2	2	2	2	2
Asymp. sig	0.000	0.000	0.000	0.000	0.000	0.002

Table S2. The major chemical constituents of *Citrus aurantium* essential oil

No.	RT	Compound name	Area (%)	R. I.	M. F.	Classification
1	4.88	α-Pinene	1.40	937	$C_{10}H_{16}$	monoterpene
2	5.82	Sabinene	0.80	974	$C_{10}H_{16}$	monoterpene
3	6.26	β-Myrcene	2.11	967	$C_{10}H_{16}$	monoterpene
4	7.24	β-Cymene	0.66	1042	$C_{10}H_{14}$	monoterpene
5	7.40	Limonene	**91.35**	1030	$C_{10}H_{16}$	monoterpene
6	8.22	Terpinolene	**1.12**	1088	$C_{10}H_{16}$	monoterpene
7	12.77	Estragole	1.70	1196	$C_{10}H_{12}O$	phenylpropene

Table S3. The major chemical constituents of *Cymbopogon schoenanthus* essential oil

No.	RT	Compound name	Area (%)	R. I.	M. F.	Classification
1	2.02	1-Octanol, 2,7-dimethyl	1.15	975	$C_{10}H_{22}O$	fatty alcohol
2	6.26	β-Myrcene	6.27	967	$C_{10}H_{16}$	monoterpene
3	9.58	Linalool	1.23	1099	$C_{10}H_{18}O$	monoterpene
4	12.16	Isocitral	2.63	989	$C_{10}H_{16}O$	monoterpene
5	12.75	Estragole	1.70	1196	$C_{10}H_{12}O$	phenylpropene
6	14.06	Neral	**38.04**	1240	$C_{10}H_{16}O$	monoterpene
7	14.52	Geraniol	4.10	998	C10$H_{18}O$	monoterpene
8	14.52	Neryl alcohol	5.33	1228	$C_{10}H_{18}O$	monoterpene
9	15.05	2,6-Octadienal, 3,7-dimethyl-, (e)-	**39.89**	1270	$C_{10}H_{16}O$	monoterpene
10	18.54	Geranyl acetate	3.38	1382	$C_{12}H_{20}O_2$	monoterpene
11	20.16	Trans-α-Bergamotene	0.47	1435	$C_{15}H_{24}$	sesquiterpene

Table S4. The major chemical constituents of *Lavandula angustifolia* essential oil

No.	RT	Compound name	Area (%)	R. I.	M. F.	Classification
1	2.02	1-Octanol, 2,7-dimethyl	0.59	965	$C_{10}H_{22}O$	fatty alcohol
2	7.23	O-cymene	0.39	1022	$C_{10}H_{14}$	phenol
3	7.43	Eucalyptol	1.69	1032	$C_{10}H_{18}O$	monoterpene
4	8.62	Linalool oxide	1.56	1124	$C_{10}H_{18}O_2$	monoterpene
5	9.61	Linalool	**30.42**	1099	$C_{10}H_{18}O$	monoterpene
6	11.65	Lavandulol	0.88	1025	$C_{10}H_{18}O$	monoterpene
7	11.85	Borneol	1.01	988	$C_{10}H_{18}O$	monoterpene
8	12.14	Terpinen-4-ol	5.72	1260	$C_{10}H_{18}O$	monoterpene
9	12.65	β-Fenchol	1.28	980	$C_{10}H_{18}O$	monoterpene

10	12.76	Estragole	1.40	1196	$C_{10}H_{12}O$	phenylpropene
11	14.45	Linalyl acetate	**42.28**	1257	$C_{12}H_{20}O_2$	monoterpene
12	15.54	Lavandulol acetate	**4.36**	1257	$C_{12}H_{20}O_2$	monoterpene
13	18.55	Geranyl acetate	**0.49**	1382	$C_{12}H_{20}O_2$	monoterpene
14	19.68	Caryophyllene	2.98	1419	$C_{15}H_{24}$	sesquiterpene
15	20.83	Cis-*β*-Farnesene	1.62	1589	$C_{15}H_{24}$	sesquiterpene
16	24.50	Caryophyllene oxide	3.33	1581	$C_{15}H_{24}O$	sesquiterpene

Table S5. The major chemical constituents of *Narcissus tazetta* essential oil

No.	RT	Compound name	Area (%)	R. I.	M. F.	Classification
1	4.36	β-pinene	0.35	974	$C_{10}H_{16}$	monoterpene
2	5.24	β-Cymene	0.11	1042	$C_{10}H_{14}$	monoterpene
3	5.35	D-Limonene	2.34	1030	$C_{10}H_{16}$	monoterpene
4	6.97	Linalool	5.51	1099	$C_{10}H_{18}O$	monoterpene
5	7.29	Phenylethyl alcohol	0.42	914	$C_8H_{10}O$	phenyl
6	8.15	β-Terpineol	0.16	1150	$C_{10}H_{18}O$	monoterpene
7	8.44	Acetic acid, phenylmethyl ester	1.35	1164	$C_9H_{10}O_2$	phenol
8	9.10	Phenylethyl acetate	0.12	1357	$C_{10}H_{12}O_2$	Phenylpropene
9	9.32	*α*-Terpineol	1.62	1260	$C_{10}H_{18}O$	monoterpene
10	9.45	Geraniol	2.04	998	$C_{10}H_{18}O$	monoterpene
11	9.91	Butanoic acid, 1-methyloctyl ester	3.50	1486	$C_{13}H_{26}O_2$	flavonoid
12	10.16	Citronellol	**7.39**	1135	$C_{10}H_{20}O$	monoterpene
13	11.66	Estragole	0.39	1196	$C_{10}H_{12}O$	phenylpropene
14	13.05	Methyl anthranilate	0.85	1320	$C_8H_9NO_2$	anthranilic acid
15	13.34	Eugenol	0.21	1357	$C_{10}H_{12}O_2$	phenylpropene
16	13.95	Geranyl acetate	0.16	1382	$C_{12}H_{20}O_2$	monoterpene
17	14.68	β-Ionone	0.14	1486	$C_{13}H_{20}O$	flavonoid
18	14.96	Caryophyllene	0.44	1419	$C_{15}H_{24}$	sesquiterpene
19	15.30	Dihydro-β-ionone	1.43	1245	$C_{13}H_{22}O$	phenol
20	15.97	Cyclamen aldehyde	0.43	1330	$C_{13}H_{18}O$	fatty acid
21	16.46	Germacrene D	0.60	1435	$C_{15}H_{24}$	polyphenol
22	16.61	Benzyldimethylcarbinyl butyrate	0.82	1370	$C_{14}H_{20}O_2$	flavonoid
23	17.06	*α*-Farnesene	0.68	1419	$C_{15}H_{24}$	sesquiterpene
24	17.32	2-Phenoxyethyl isobutyrate	0.63	1346	$C_{12}H_{16}O_3$	phenol ether
25	19.08	Diethyl phthalate	**13.35**	1594	$C_{12}H_{14}O_4$	phthalic acid
26	19.76	Cedrenol	1.31	1581	$C_{15}H_{24}O$	sesquiterpene
27	19.93	Methyl-β-ionone	8.00	1480	$C_{14}H_{22}O$	sesquiterpene
28	20.22	Dihydro-3-oxo-β-ionol	0.21	1320	$C_{13}H_{22}O_2$	phenol
29	20.52	Methyl dihydrojasmonate	**11.36**	1649	$C_{13}H_{22}O_3$	ketone
30	20.85	Cis-3-Hexenyl salicylate	1.16	1211	$C_{13}H_{16}O_3$	phenol
31	21.32	β-Ionone	2.55	1456	$C_{14}H_{22}O$	sesquiterpene
32	21.89	Trans-farnesol	0.22	1522	C15H26O	sesquiterpene
33	22.50	Cinnamaldehyde, *α*-hexyl	3.04	1427	$C_{15}H_{20}O$	phenol
34	22.90	Acetyl cedrene	4.39	1645	$C_{17}H_{26}O$	ketone
35	23.65	6,7-diethyl-1,1,4,4-tetramethyl-2,3-dihydronaphthalene	4.74	1861	$C_{18}H_{28}$	phenol
36	24.49	7-Acetyl-6-ethyl-1,1,4,4-tetramethyltetralin	**17.98**	1851	$C_{18}H_{26}O$	phenol

Table S6. The major chemical constituents of *Origanum majorana* essential oil

No.	RT	Compound name	Area (%)	R. I.	M. F.	Classification

1	4.71	Bicyclo[3.1.0]hex-2-ene, 4-methyl-1-(1-methylethyl)-	0.65	974	$C_{10}H_{16}$	monoterpene
2	4.87	α-Pinene	1.36	937	$C_{10}H_{16}$	monoterpene
3	5.82	Sabinene	7.59	974	$C_{10}H_{16}$	monoterpene
4	6.26	α-Myrcene	1.21	1030	$C_{10}H_{16}$	monoterpene
5	6.70	α-Phellandrene	0.33	955	$C_{10}H_{16}$	monoterpene
6	7.00	α-Terpinene	**7.82**	974	$C_{10}H_{16}$	monoterpene
7	7.23	M-Cymene	3.13	1042	$C_{10}H_{14}$	monoterpene
8	7.37	D-Limonene	4.77	1030	$C_{10}H_{16}$	monoterpene
9	8.23	γ-Terpinene	**11.69**	1060	$C_{10}H_{16}$	monoterpene
10	8.62	Cis-4-Thujanol	**19.70**	1090	$C_{10}H_{18}O$	monoterpene
11	9.06	Terpinolene	2.85	1088	$C_{10}H_{16}$	monoterpene
12	10.91	1-Terpinenol	0.86	1099	$C_{10}H_{18}O$	monoterpene
13	12.16	Terpinen-4-ol	**23.50**	1260	$C_{10}H_{18}O$	monoterpene
14	12.65	α-Terpinyl acetate	4.52	2110	$C_{10}H_{18}O$	monoterpene
15	13.11	Piperitol	0.43	1190	$C_{10}H_{18}O$	monoterpene
16	14.42	Linalyl acetate	2.83	1257	$C_{12}H_{20}O_2$	monoterpene
17	19.68	Caryophyllene	3.83	1419	$C_{15}H_{24}$	sesquiterpene
18	22.00	Bicyclogermacrene	2.09	1419	$C_{15}H_{24}$	sesquiterpene
19	24.40	Sathulenol	0.24	1581	$C_{15}H_{24}O$	sesquiterpene

Table S7. The major chemical constituents of *Pelargonium graveolens* essential oil

No.	RT	Compound name	Area (%)	R. I.	M. F.	Classification
1	5.36	2-Propanol, 1,1'-oxydi	7.26	1046	$C_6H_{14}O_3$	phenol
2	5.88	1-Propanol, 2-(2-hydroxypropoxy)	**15.77**	1046	$C_6H_{14}O_3$	phenol
3	6.69	Malonic acid	1.85	345	C_3H_4O	malonate
4	6.94	Linalool	0.29	1099	$C_{10}H_{18}O$	monoterpene
5	7.45	Phenylethyl alcohol	**35.51**	914	$C_8H_{10}O$	phenyl
6	8.29	Menthone	0.09	1135	$C_{10}H_{18}O$	monoterpene
7	8.45	Acetic acid, phenylmethyl ester	3.17	1164	$C_9H_{10}O_2$	phenol
8	8.76	Benzenepropanoic acid, methyl ester	1.61	1224	$C_9H_{10}O_2$	phenol
9	9.86	α-Citronellol	0.13	1135	$C_{10}H_{20}O$	fatty alcohol
10	10.15	β-Citronellol	**7.60**	1135	$C_{10}H_{20}O$	monoterpene
11	10.25	3-Phenylpropanol	1.66	1340	$C_9H_{12}O$	phenol
12	10.75	Geraniol	3.08	998	$C_{10}H_{18}O$	monoterpene
13	12.33	Benzeneethanol, α,α-dimethyl, acetate	1.51	1298	$C_{12}H_{16}O_2$	flavonoid
14	13.23	Citronellol acetate	0.17	1298	$C_{12}H_{22}O_2$	monoterpene
15	15.04	β-Ionone	3.13	1486	$C_{13}H_{20}O$	flavonoid
16	16.29	2(E)-Decenal	1.66	960	$C_{10}H_{18}O$	aldehyde
17	16.42	Trans-β-Ionone	1.82	1486	$C_{13}H_{20}O$	flavonoid
18	16.83	9-Octadecenoic acid (z)	0.15	2175	$C_{18}H_{34}O_2$	Oleic acid
19	18.03	Rose acetate	3.24	1094	$C_{10}H_9Cl_3O_2$	fatty acid
20	20.39	Methyl dihydrojasmonate	0.62	1649	$C_{13}H_{22}O_3$	ketone
21	25.89	Benzeneacetic acid, 2-phenylethyl ester	**9.68**	1909	$C_{16}H_{16}O_2$	Phenylacetic acid

Table S8. The major chemical constituents of *Punica granatum* essential oil

No.	RT	Compound name	Area (%)	R. I.	M. F.	Classification
1	13.27	Tetradecane, 2,6,10-trimethyl-	2.24	1780	$C_{17}H_{36}$	alkane
2	15.20	Ambreinolide	0.59	1645	$C_{17}H_{28}O_2$	α-keto acid

3	15.73	3,7,11-Trimethyl-1-dodecanol	3.40	1755	$C_{17}H_{36}$	alkane
4	15.73	1-Tetradecanol	0.45	1386	$C_{14}H_{30}O$	fatty alcohol
5	16.10	Docosane	1.88	1876	$C_{22}H_{46}$	alkanes
6	16.27	Tetradecane, 2,6,10-trimethyl	3.34	1780	$C_{17}H_{36}$	alkane
7	17.62	2-Hexadecanol	0.38	1890	$C_{16}H_{34}O$	fatty alcohol
8	18.48	Heptacosane	0.89	1986	$C_{27}H_{56}$	alkane
9	18.64	3-methyl-pentadecane	1.29	1925	$C_{16}H_{34}$	alkane
10	18.97	Diethyl Phthalate	**27.97**	1594	$C_{12}H_{14}O_4$	Phthalic acid
11	19.32	14-β-H-pregna	**42.39**	2520	$C_{21}H_{36}$	fatty acid
12	20.33	1-(4-Bromobutyl)-2-piperidinone	2.99	1635	$C_9H_{16}BrNO$	fatty acid
13	20.86	Hexadecahydropyrene	3.85	1502	$C_{16}H_{26}$	fatty acid
14	21.97	Isocritonilide	7.50	1415	$C_{15}H_{20}O_2$	sesquiterpene
15	23.27	2-Methyl-E,E-3,13-octadecadien-1-ol	**1.20**	٢١٠٤	$C_{19}H_{36}O$	fatty acid

Table S9. The major chemical constituents of *Ricinus communis* essential oil

No.	RT	Compound name	Area (%)	R. I.	M. F.	Classification
1	2.02	Cyclobutane, 1,1-dimethyl-2-octyl	**31.33**	913	$C_{14}H_{28}$	cycloalkane
2	7.35	β-Pinene	7.27	937	$C_{10}H_{16}$	monoterpene
3	7.41	Eucalyptol	7.30	1032	$C_{10}H_{18}$	monoterpene
4	9.60	2-Methyl-1-Hexadecanol	4.10	1830	$C_{17}H_{36}O$	alkane
5	10.95	Chamazulene	6.44	1420	$C_{14}H_{16}$	phenol
6	12.15	Terpinen-4-ol	6.63	1260	$C_{10}H_{18}O$	monoterpene
7	12.80	Estragole	**24.95**	1196	$C_{10}H_{12}O$	phenylpropene
8	33.82	Oxiraneundecanoic acid, 3-pentyl-, methyl ester, cis	4.77	٢١75	$C_{19}H_{36}O$	fatty acid
9	36.65	Hexadecanoic acid, trimethylsilyl ester	3.85	2215	$C_{19}H_{40}O_2Si$	palmitic acid
10	37.84	9-Octadecenoic acid (z)	3.36	2175	$C_{18}H_{34}O_2$	oleic acid

Table S10. The major chemical constituents of *Rosmarinus officinalis* essential oil

No.	RT	Compound name	Area (%)	R. I.	M. F.	Classification
1	2.02	Cyclobutane, 1,1-dimethyl-2-octyl	**0.47**	913	$C_{14}H_{28}$	cycloalkane
2	4.63	Tricyclene	**1.63**	937	$C_{10}H_{16}$	monoterpene
3	4.87	α-Pinene	**20.85**	937	$C_{10}H_{16}$	monoterpene
4	5.25	Camphene	8.04	952	$C_{10}H_{16}$	monoterpene
5	5.93	β-Pinene	6.69	988	$C_{10}H_{16}$	monoterpene
6	6.25	β-Myrcene	0.42	967	$C_{10}H_{16}$	monoterpene
7	6.77	3-Carene	3.53	952	$C_{10}H_{16}$	monoterpene
8	6.94	1,4-Cineole	0.32	1099	$C_{10}H_{18}O$	monoterpene
9	7.23	O-Cymene	4.49	1022	$C_{10}H_{14}$	phenol
10	7.42	Eucalyptol	**20.45**	1032	$C_{10}H_{18}$	monoterpene
11	8.22	γ-Terpinene	2.96	976	$C_{10}H_{16}$	monoterpene
12	9.59	Linalool	1.54	1099	$C_{10}H_{18}O$	monoterpene
13	10.95	Camphor	**14.65**	937	$C_{10}H_{16}O$	monoterpene
14	11.54	Borneol	11.32	1150	$C_{10}H_{18}O$	monoterpene
15	12.13	Terpinen-4-ol	1.98	1260	$C_{10}H_{18}O$	monoterpene
16	12.64	α-Terpineol	1.64	1260	$C_{10}H_{18}O$	monoterpene
17	12.78	Estragole	0.69	1196	$C_{10}H_{12}O$	phenylpropene

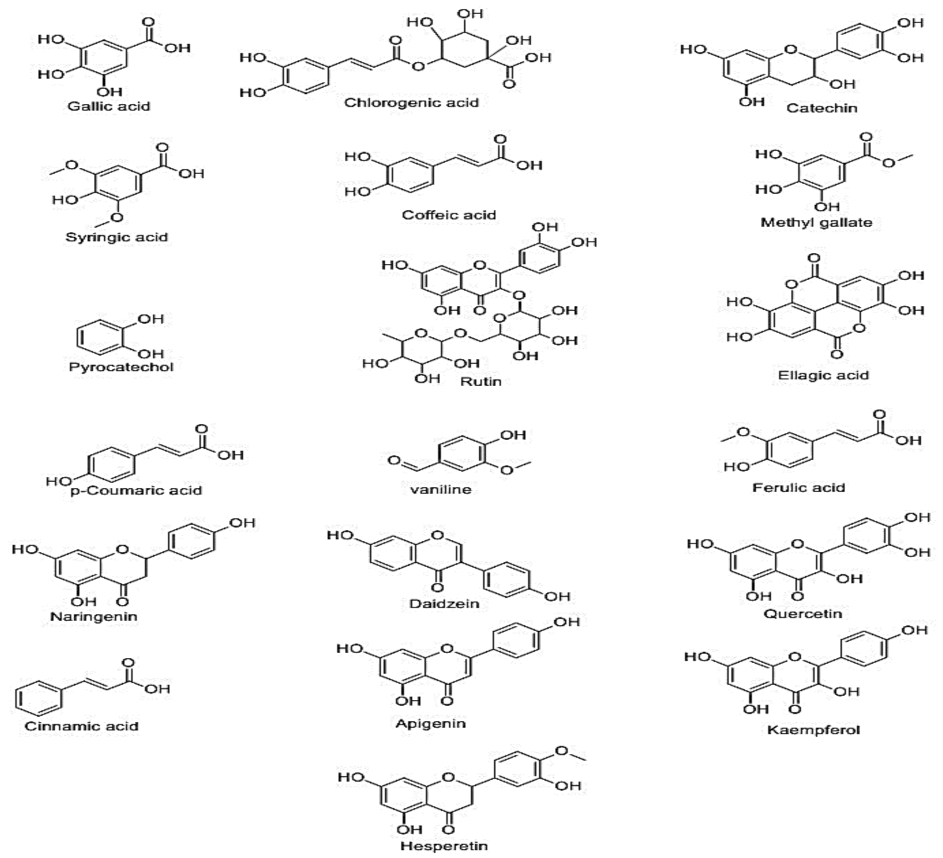

Figure S1. Structure of the tested polyphenol active components by HPLC.

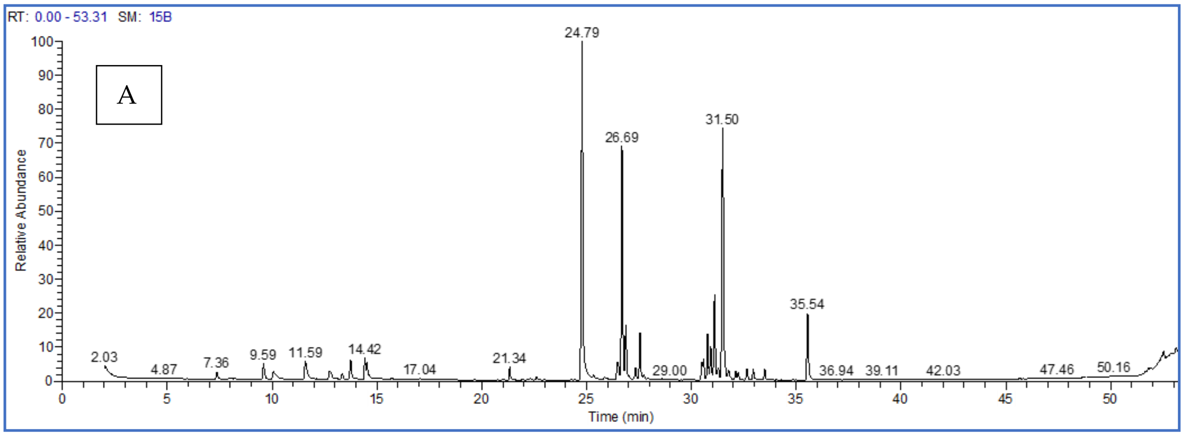

Figure S2. GC chromatogram of *Lonicera caprifolium* (a) and *Pogostemon cablin* (b) essential oils.

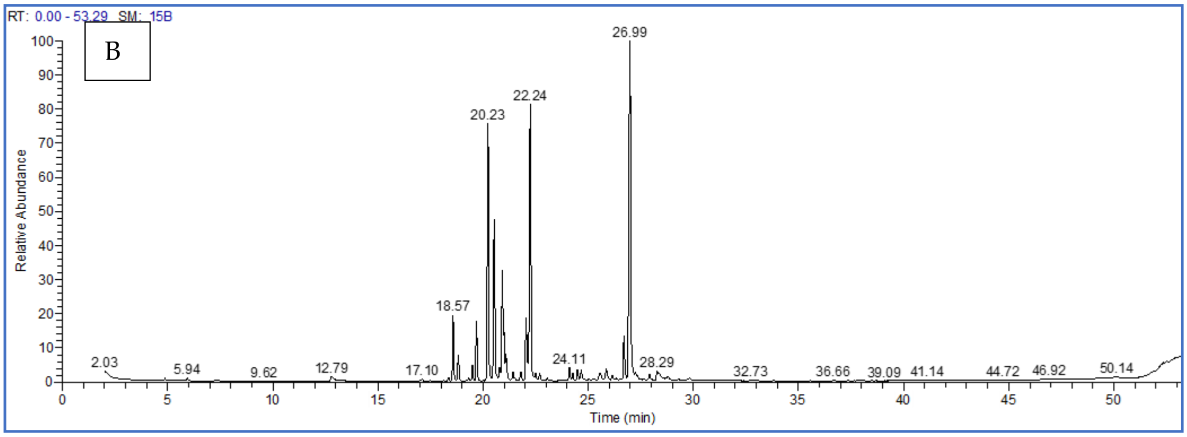

Supplement: Supplementary file 1 [file plants-12-03682-s001.zip › plants-2649822-SI.pdf]
